# Growing Different *Lactuca* Genotypes Aeroponically within a Tropical Greenhouse—Cool Rootzone Temperatures Decreased Rootzone Ethylene Concentrations and Increased Shoot Growth

**DOI:** 10.3389/fphys.2016.00405

**Published:** 2016-09-13

**Authors:** Tsui-Wei Choong, Jie He, Sing K. Lee, Ian C. Dodd

**Affiliations:** ^1^Natural Sciences and Science Education, National Institute of Education, Nanyang Technological UniversitySingapore, Singapore; ^2^Lancaster Environment Centre, Lancaster UniversityLancaster, UK

**Keywords:** spray intervals, SLA, chlorophyll, carotenoid, NPQ, qP, ETR

## Abstract

Temperate crops cannot grow well in the tropics without rootzone cooling. As cooling increased production costs, this experiment aimed to study the growth of various *Lactuca* genotypes and propose possible ways of reducing these costs, without compromising productivity. A recombinant inbred line (RIL) of lettuce and its parental lines (*L. serriola* and *L. sativa* “Salinas”) were grown aeroponically in a tropical greenhouse under 24°C cool (C) or warm fluctuating 30–36°C ambient (A) rootzone temperature (RZT). Their roots were misted with Netherlands standard nutrient solution for 1 min, at intervals of either 5 min (A5, C5) or 10 min (A10, C10) in attempting to reduce electricity consumption and production costs. Lower mortality and higher productivity were observed in all genotypes when grown in C-RZT. Higher shoot fresh weight was observed under C5 than C10, for the RIL and *L. serriola*. Since “Salinas” had similar shoot fresh weight at both C-RZ treatments, this may indicate it is more sensitive to RZT than water availability. Under A-RZ treatments, higher carotenoid content, with correspondingly higher nonphotochemical quenching, was observed in A10 for the RIL and “Salinas.” Further, total chlorophyll content was also highest at this RZ treatment for the RIL though photochemical quenching was contrastingly the lowest. Cumulatively, productivity was compromised at A10 as the RIL seemed to prioritize photoprotection over efficiency in photosynthesis, under conditions of higher RZT and lower water availability. Generally, higher RZ ethylene concentrations accumulated in A10 and C10 than A5 and C5, respectively—probably due to spray frequency exerting a greater effect on RZ ethylene accumulation than RZT. In the C5 RZ treatment, lowest RZ ethylene concentration corresponded with highest shoot fresh weight. As such, further research on ethylene (in)sensitivity and water use efficiency could be conducted to identify *Lactuca* cultivars that are better suited for growth in the tropics, so as to allay production costs with reduced cooling and spray intervals.

## Introduction

As roots are more thermosensitive than shoots (Tachibana, 1982; Thompson et al., 1998; Sakamoto and Suzuki, 2015), temperate and subtropical crops have been successfully grown in a tropical greenhouse by cooling only their roots (Lee et al., 1994; Choong, 1998; He and Lee, 1998; He et al., 2001). Taking advantage of the innately high specific heat capacity of water, aeroponic systems use small volumes of chilled nutrient solution to lower RZTs to conditions that are ideal for the proliferation of these temperate and subtropical crops (Lee, 1993). However, this cooling inflates production costs. As such, this research ventures to better understand the growth characteristics of different *Lactuca* genotypes in attempting to lower production costs without compromising productivity.

*Lactuca serriola* L., the wild-type ancestor of cultivated lettuce (*Lactuca sativa* L.) (Durst, 1929; Kesseli et al., 1991; Harlan, 1992) is a drought tolerant winter annual (Werk and Ehleringer, 1985) well-adapted to Europe (Kesseli et al., 1991), nontropical parts of Eurasia and North Africa (Kirpicznikov, 1964; Jeffrey, 1975; Ferakova, 1976), North America, and South Africa (Zohary, 1991). As cultivated lettuce is a temperate plant, growing it under tropical conditions decreased head biomass and quality (He and Lee, 1998; He et al., 2001; He, J. et al., 2009; Choong et al., 2013). He and Lee (1998) obtained lettuce that was at least four times heavier when grown in C-RZT of 15–25°C than A-RZT (26–41°C), in a tropical greenhouse where aerial temperatures reached a maximum of 41°C at midday. He, J. et al. (2009) similarly reported increased total leaf number and shoot fresh mass of lettuce plants grown in 20°C-RZT when compared to plants in A-RZT. The low productivity of lettuce growing in such warm conditions has been correlated with reduced root growth (Kaspar and Bland, 1992; He and Lee, 1998; Choong et al., 2013), where the roots cannot adequately supply water and nutrients to the shoot (He et al., 2001; Dodd, 2005), thereby limiting photosynthesis (He et al., 2001). Decreased root fresh weight also correlated with root morphological traits including decreased total root length, root surface area, and number of root tips (i.e., branching) in A-RZT (Choong et al., 2013). These poorly developed root systems affect shoot growth as root-sourced signals that regulate shoot growth are transmitted via the xylem (Freundl et al., 1998; Dodd, 2005). Greater temperature tolerance of some accessions of *L. serriola* (Argyris et al., 2008) offers the possibility of minimizing shoot growth inhibition caused by A-RZT.

Salisbury and Ross (1992) reported that high air temperatures deleteriously impaired photosynthesis, while high irradiance also decreased photosynthetic rates (He et al., 2001; Barker et al., 2002; He and Lee, 2004) since excess photon energy caused photoinactivation of photosystem II (Björkman and Powles, 1984). As a result, higher nonphotochemical quenching, demonstrating greater amounts of energy being dissipated as heat (He et al., 2015), and lower electron transport rate were observed when growing lettuce in A-RZT than 20°C-RZT (He and Lee, 2004). Chronic photoinhibition in A-RZT plants was associated with a 20% reduction in chlorophyll content (He, 2009) and such reductions have been proposed to provide protection by reducing photon absorption (Verhoeven et al., 1997). Thus, plants grown at aerial temperatures of 25°C were less green and had thinner leaves than those grown at 15°C (Dale, 1965). Chlorophyll loss has further been related to environmental stress, where variations in chlorophyll/carotenoid ratios have been reported to be good indicators of plant stress (Hendry and Price, 1993). Further, Rubisco—the key photosynthetic enzyme—and other carbon metabolism enzymes were also temperature-sensitive, thereby impacting growth (Berry and Raison, 1981). As such, high RZT and light intensity compromise productivity by affecting various physiological mechanisms.

Plants synthesize ethylene, a gaseous plant hormone, and release it into the atmosphere during their normal growth and development. Regulating root growth (Ruzicka et al., 2007), low exogenous ethylene concentrations also stimulate vegetative growth (Pierik et al., 2006). However, enhanced ethylene concentrations have frequently been measured in plants exposed to environmental stresses (Abeles et al., 1992; Morgan and Drew, 1997; Lin et al., 2009), and ethylene can inhibit stem elongation (Abeles et al., 1992) and leaf expansion (Lee and Reid, 1997) without directly affecting leaf gas exchange (Pallaghy and Raschke, 1972; Woodrow et al., 1989) or photosynthesis (Abeles et al., 1992). Moreover, ethylene decreased net carbon gain indirectly by inducing leaf epinasty which decreased light interception (Woodrow and Grodzinski, 1989; He, C. J. et al., 2009). Under ambient conditions, increasing ethylene concentrations were correlated with decreased carbon dioxide assimilation and growth (He, C. J. et al., 2009). Indeed, applying ACC to aeroponically grown lettuce (*Lactuca sativa* cv. Baby Butterhead) at A-RZT in a tropical greenhouse decreased stomatal conductance, leaf relative water content, photosynthetic CO_2_ assimilation, shoot and root biomass compared with plants grown at 20°C-RZT (Qin et al., 2007). Since the immediate precursor of ethylene (ACC: 1-aminocyclopropane-1-carboxylic acid) is synthesized in the roots (Dodd, 2005) and ACC synthase activity increases with temperature (Ainscough et al., 1992), it is reasonable to suggest that higher amounts of endogenous ethylene may limit root (and shoot) growth at A-RZT. This experiment examines RZ ethylene accumulation in relation to the growth and photosynthetic characteristics of lettuce, whilst varying spray intervals and RZT in the aeroponic system, as an approach toward reducing production costs.

## Materials and methods

### Plant materials and culture methods

Seeds of maternal *L. sativa* L. “Salinas” and paternal *L. serriola* accession UC96US23 (Argyris et al., 2005), together with an F_10_ RIL were germinated on wet filter paper in a petri dish. This thermotolerant RIL was selected based upon previous research carried out (Choong et al., 2013). Five days after germination, seedlings were inserted into polyurethane cubes and left to acclimatize in ambient tropical greenhouse conditions for 7 days before being transplanted into the aeroponic system (Lee, 1993). They were grown in either A-RZT (29–39°C) or C-RZT (21.5–28.5°C). Roots were misted for 1 min with full strength Netherlands Standard Nutrient Solution (2.2 mS, pH 6.5; Douglas, 1985), at 5 or 10 min intervals, giving rise to four experimental conditions: A5, A10, C5, and C10. Shoots were exposed to fluctuating ambient temperatures of 25–39°C and 70–95% relative humidity, under 100% prevailing solar radiation, with maximum photosynthetic photon flux density (PPFD) of 1000 μmol photon m^−2^ s^−1^.

### Measurement of RZT

RZT was tracked, at 20 min intervals, across the 28-day growing period using a temperature probe (SL52T, Signatrol) that was left in the RZ of the growing trough. Data was then downloaded using its accompanying software TempIt-Pro (Version 4.1.41, Signatrol) and plotted in Microsoft Excel (Version 14.0, 2010).

### Measurements of mortality and growth parameters

Some plants succumbed following transplant and the number of plants remaining was counted 28 days after transplanting, and % mortality was calculated. Three or more plants of each genotype were harvested 35 days after transplanting and their total shoot and root fresh weights (FW) per trough were measured and means calculated. Specific leaf area (SLA) was calculated by dividing the area of 10 1-cm-diameter leaf discs with their dry weights after drying for 5 days in a 65°C oven. Three or more replicates were used.

### Measurements of photosynthetic pigments

Four 1-cm-diameter leaf discs obtained from newly expanded leaves from four different plants were soaked in 1.5 ml N,N-dimethylformamide for 48 h in the dark, at 4°C. Absorption of 4 replicates was read at 480, 647, and 664 nm, using a spectrophotometer (UV-2550, Shimadzu, Japan). Concentrations for chlorophyll (Chl) a, Chl b, and carotenoids were calculated (Wellburn, 1994).

### Measurements of photochemical light use efficiency

Leaves were harvested at 0900 h for Chl fluorescence analysis, where nonphotochemical quenching (NPQ), photochemical quenching (qP) and electron transport rate (ETR) of four detached newly expanded leaves from four different plants were measured at 25°C in the laboratory, using the Imaging-PAM Chlorophyll Fluorometer (Walz, Effeltrich, Germany) (He et al., 2011).

### Measurements of RZ ethylene accumulation

Each plant genotype for each RZ-treatment was grown within individual troughs and the ethylene gas that had accumulated in the RZ space of plants of the entire trough was extracted between spray cycles, at 1300 h on a sunny day, using a 500 ml gas syringe (Hamilton, USA). Air samples were transferred into their respective 1 L gas sampling bags (Sigma-Aldrich) in the greenhouse and brought back to the laboratory to allow air temperatures within the bags to acclimatize to room temperatures (28°C) prior to sampling. The bags were connected to the EASI-1 portable ethylene analyzer (Absoger, France), via a 15 cm long tube filled with silica gel, and left to stabilize until consistent readings (±10% variation) were obtained. At least three consecutive readings were taken per sample gas bag.

### Statistical analysis

A mixed-model nested analysis of variance (ANOVA) was performed using SPSS (Version 20, 2011) to test for significant effects of variation between genotypes and their response to the four RZ-treatments (A5, A10, C5, and C10) using *post-hoc* Tukey's pairwise tests, at a significance level of α = 0.05.

## Results

### RZT, mortality, and growth parameters

**Figure 1:**
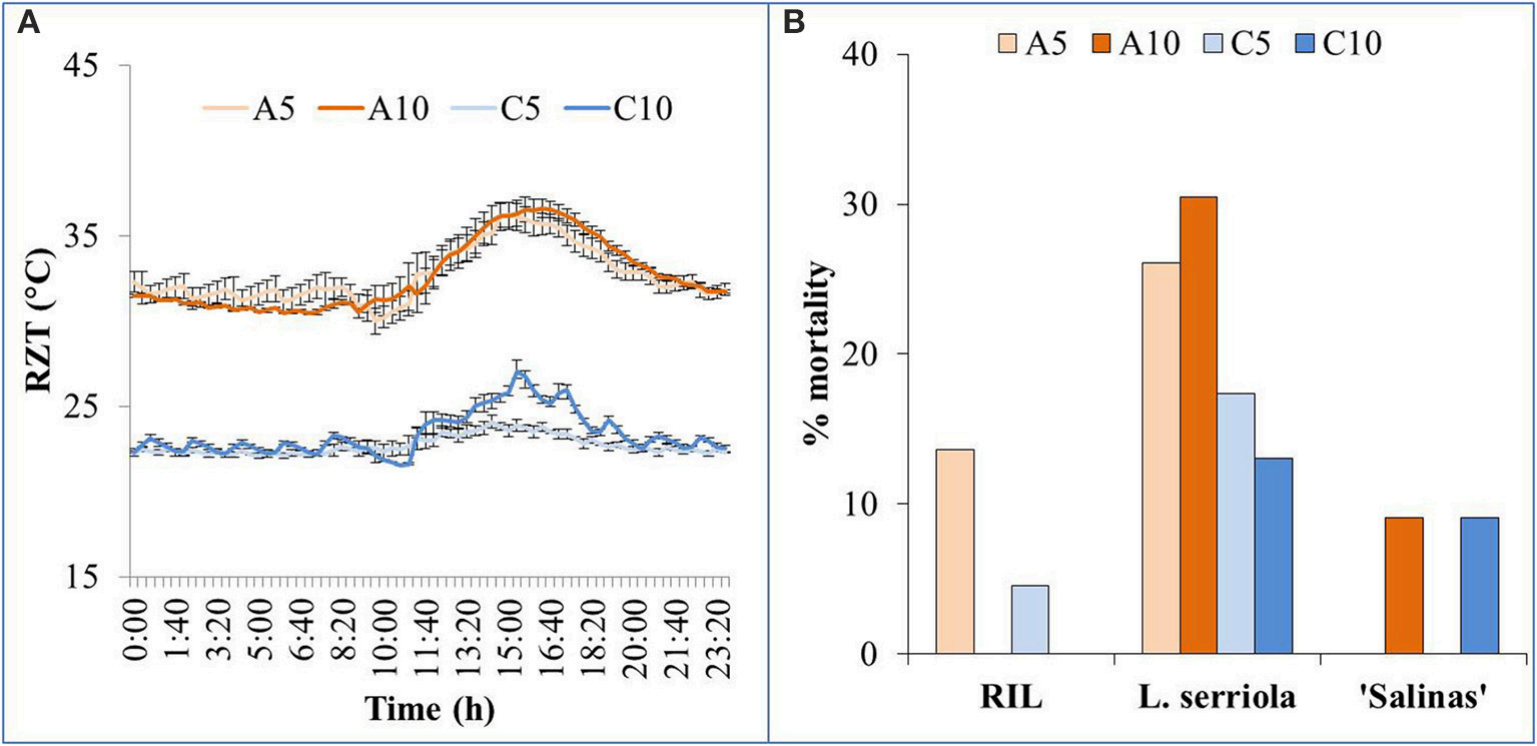
**(A)** Range and variability of RZT of the four experimental conditions (A5, A10, C5, and C10) for the 3 plant types, with SE bars showing the variability across the 28-day growing period. **(B)** 22 seedlings of all plant genotypes were grown in the 4 RZ treatments and mortality was observed. Graph shows the % mortality of each genotype in each of the 4 RZ treatments, at harvest, 28 days after transplanting.

Plants that were growing in A5 and A10 were exposed to very similar RZT ranges (Figure 1A), throughout the 28-day growth period. Temperature range for A5 and A10 were 29–37°C and 29–39°C, respectively. Plants in C5 and C10 were also exposed to similar RZTs (Figure 1A), fluctuating mostly about 23°C. Temperature range for C5 and C10 were 21.5–25.5°C and 21.5–28.5°C, respectively. RZT in C10 was much higher than C5, between 1100 and 2000 h. The temperature in C10 even exceeded 25°C for a period of 4 h from 1340 to 1740 h (Figure 1A).

Some mortality was observed within the seedlings that were transplanted (Figure 1B). Mortality was observed only in A5 and C5 RZ treatments in the RIL, and A10 and C10 RZ treatments in “Salinas.” However, *L. serriola* exhibited mortality in all RZ treatments, with more than 25% mortality in A5 and A10 (Figure 1B). Mortality for *L. serriola* was, indeed, higher in A- than C-RZT.

**Figure 2:**
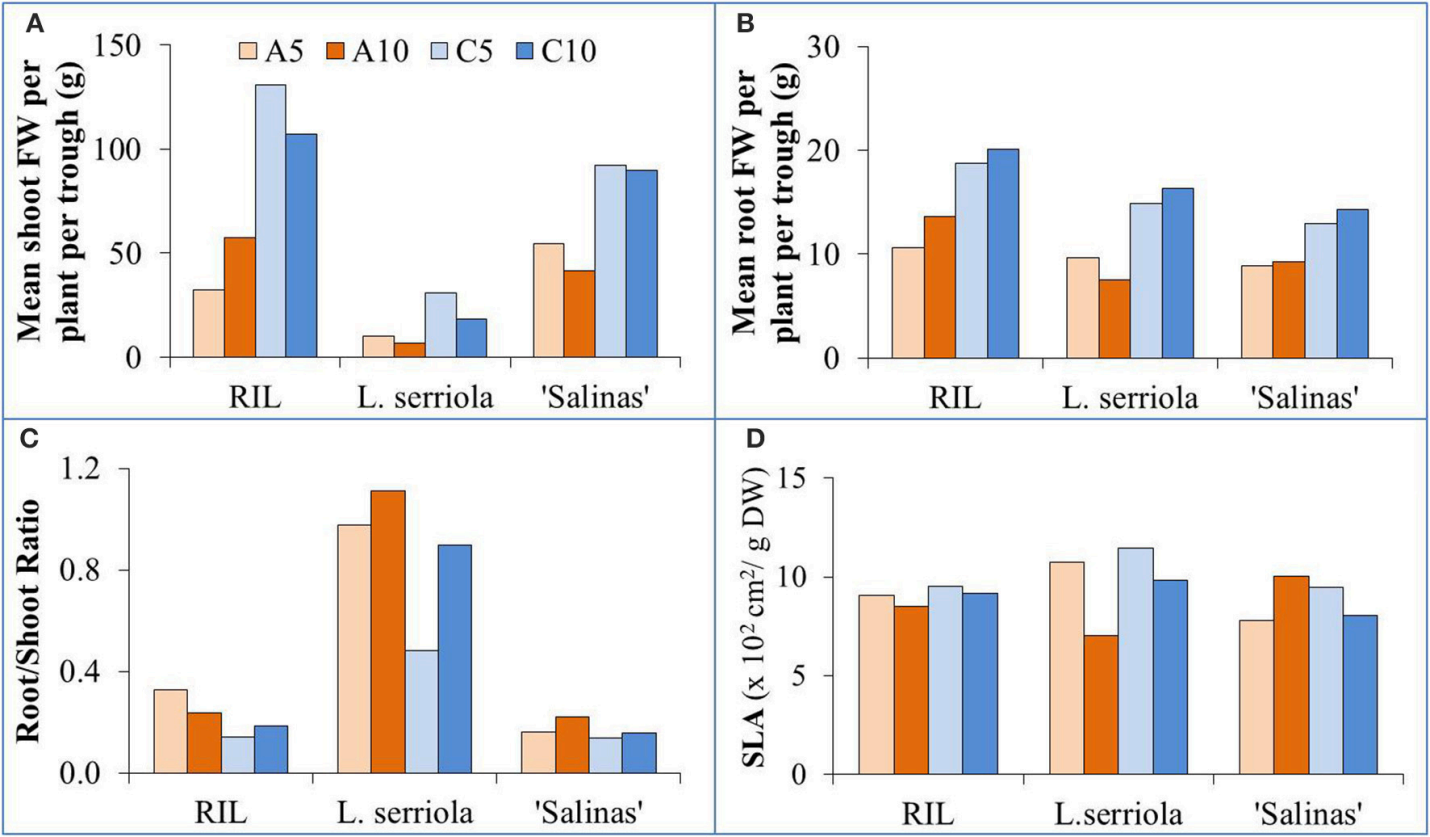
**Growth parameters of (A) mean shoot FW per plant per trough, (B) mean root FW per plant per trough, (C) root/ shoot ratio, and (D) SLA for the four experimental conditions (A5, A10, C5, and C10) for the 3 genotypes**. Cumulative shoot and root FWs of all plants in each growing trough were measured and their FWs were normalized due to the different numbers of plants sampled.

In general, the RIL was the largest plant especially in C-RZT conditions, exceeding 100 g in shoot FW (Figure 2A), while *L. serriola* was the smallest with less than 30 g for all treatments. Shoot FW was consistently higher in C-RZT than A-RZT for all plant types (Figure 2A). In A-RZT, higher shoot FW was found in the RIL at A10 rather than at A5. Highest root FW for all genotypes was in C10 (Figure 2B). Similar to the changes in shoot FW, higher root FWs were found in C-RZT than A-RZT. For root/shoot ratios (Figure 2C), *L. serriola* had the highest ratios in A-RZ treatments. For RIL and *L. serriola*, SLA was higher at C5 than A5, and also at C10 than A10 (Figure 2D), whereas it was reversed in “Salinas” at the A-RZ treatments. The lowest SLA for *L. serriola* was in A10 while “Salinas” had its highest SLA in the same treatment (Figure 2D). The RIL had similar SLA values under all treatments.

### Pigment content and photochemical light use efficiency

**Figure 3:**
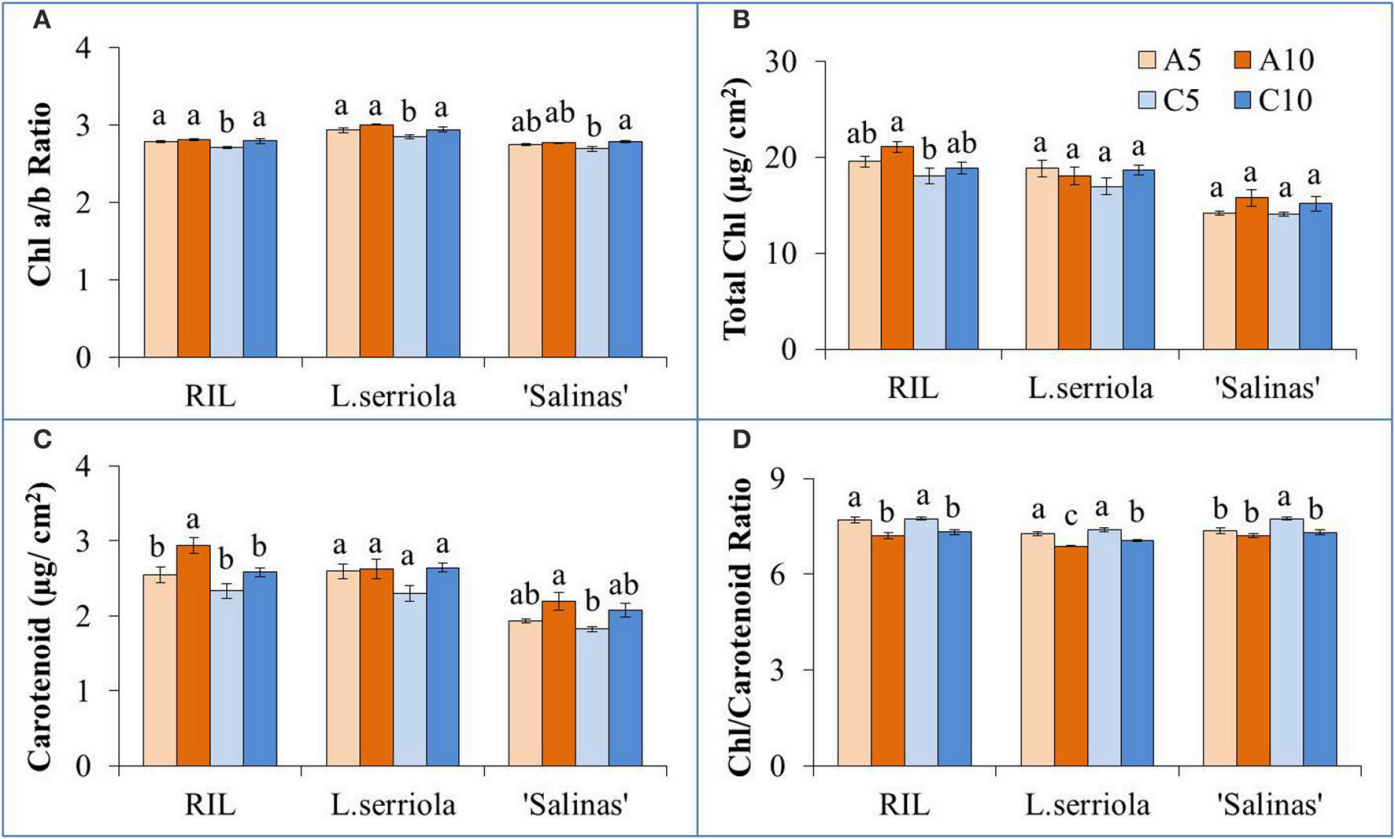
**(A)** Chlorophyll a/b ratio, **(B)** total chlorophyll concentration, **(C)** carotenoid concentration, and **(D)** chlorophyll/ carotenoid ratio of *L. serriola*, “Salinas” and RIL grown in A5, A10, C5, and C10. Each bar graph is the mean of 4 measurements from at least 4 different plants (*n* ≥ 4). Vertical bars represent standard errors. Different letters above the bar graphs denote statistical differences (*p* < 0.05) as determined by Tukey's multiple comparison test.

For all genotypes and RZ treatments, Chl a/b ratio was close to 3 (Figure 3A). Significantly lower ratios were obtained for C5-RZ treatment (*p* < 0.001) for all genotypes. RZ treatment only affected total Chl concentrations in the RIL (Figure 3B) though there was significant difference between genotypes (*p* < 0.05), with lowest total Chl in “Salinas” and highest in the RIL. A10-RZ treatment also resulted in significantly higher total Chl than C5-RZ treatments (*p* < 0.005) in the RIL. There was no significant difference in the carotenoid content of *L. serriola* across all RZ treatments (Figure 3C) but was significantly highest for A10 in the RIL (*p* < 0.05). The Chl/carotenoid ratio was significantly higher for C5 in all genotypes (*p* < 0.005, Figure 3D). Although there was no significant difference in the total Chl and carotenoid content of *L. serriola* between RZ treatments, the A10-RZ treatment had the lowest Chl/carotenoid ratio (*p* < 0.05) across the RZ treatments (Figures 3B–D).

**Figure 4:**
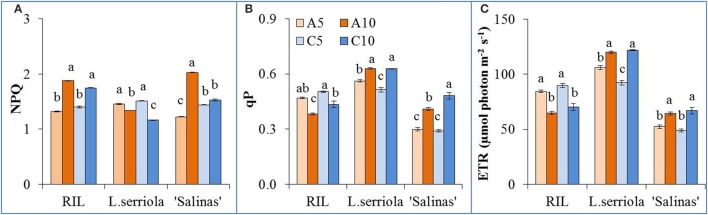
**Photochemical light use efficiency measurements of *L. serriola*, “Salinas” and RIL grown in A5, A10, C5, and C10. (A)** Nonphotochemical quenching (NPQ), **(B)** photochemical quenching (qP) and **(C)** electron transport rate (ETR) at PPFD of 605 μmol photon m^−2^ s^−1^ are shown. Each bar graph is the mean of 4 measurements from 4 different plants (*n* = 4). Vertical bars represent standard errors. Different letters above the bar graphs denote statistical differences (*p* < 0.05) as determined by Tukey's multiple comparison test.

All 3 genotypes exhibited slightly different NPQ behavior under the different RZ treatments (Figure 4A). NPQ was significantly higher for the RIL in A10 and C10 than A5 and C5. However, the reverse is observed in *L. serriola* where C10 treatment resulted in even significantly lower NPQ than A10, with both lower than the A5 and C5 treatments (Figure 4A). Conversely, qP for the RIL in A10 and C10 was significantly lower than that of A5 and C5 (Figure 4B). The qP for *L. serriola* was also inverse to its NPQ behavior. However, the qP for “Salinas” behaved similarly to that of *L. serriola* in that qP at A10 and C10 are significantly higher than that for A5 and C5 (Figure 4B). ETR for all 3 plants are closely correlated to their qP values where the RIL had higher ETR values at A5 and C5 while *L. serriola* and “Salinas” had higher values for A10 and C10 (Figure 4C).

### Accumulated RZ ethylene concentrations

**Figure 5:**
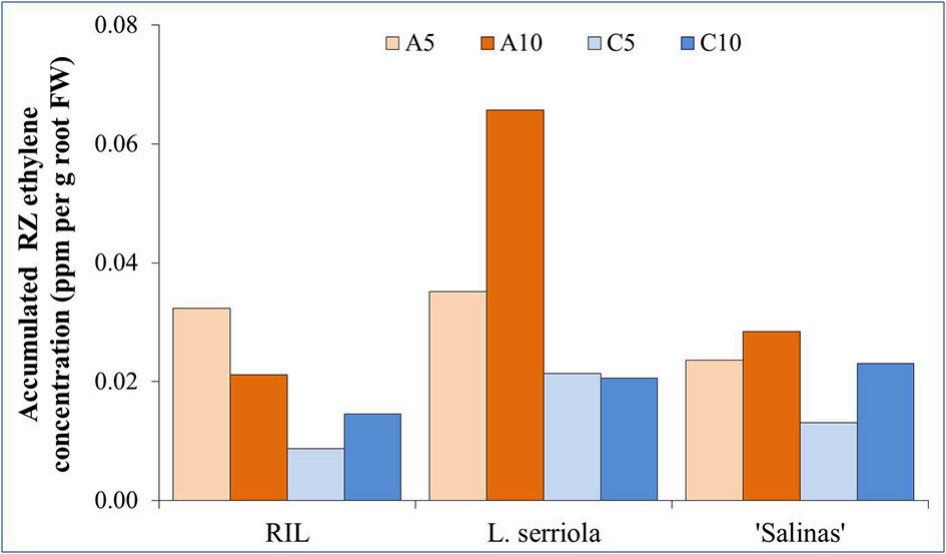
**Accumulated RZ ethylene concentration per unit root FW, measured at 1300 h on a sunny day, 28 days after transplanting into an aeroponic system**. *L. serriola*, “Salinas” and the RIL were planted in four different experimental conditions of A5, A10, C5, and C10. Each bar graph is the mean of 3 consecutive measurements for each growing condition (*n* = 3).

In general, higher concentrations of RZ ethylene accumulated in A-RZT than C-RZT (Figure 5). Highest RZ ethylene per unit root FW accumulated in A10-RZ treatment for *L. serriola*, double that of the highest values of “Salinas” and the RIL. Across treatments, “Salinas” also accumulated more RZ ethylene in the A10-RZ treatment (Figure 5). However, the A5-RZ treatment accumulated more RZ ethylene in the RIL. The lowest RZ ethylene accumulated in C5 for the RIL and “Salinas” but in *L. serriola* there was little difference between the C-RZ treatments (Figure 5).

## Discussion

Lettuce production was possible, even at particularly high air temperatures of up to 41°C, as long as their roots were cooled (He and Lee, 1998; He et al., 2001). For this experiment, all plants were exposed to fluctuating tropical air temperatures while their RZTs were maintained within two different ranges (Figure 1A). The A-RZT for A5 and A10 treatments were similar (and ranged between 29–37°C and 29–39°C, respectively) but the two C-RZ treatments differed, up to almost 4°C, for 8 h (Figure 1A) in the latter half of the day. More frequent spraying improved growth of all genotypes at C-RZ treatments (Figure 2A). For all genotypes, shoot growth was greater under C-RZT than A-RZT. Under A-RZ conditions, the effects of spray frequency varied between genotypes. Shoot growth of *L. serriola* and “Salinas” fared better in A5 than A10, unlike the RIL which had better growth at A10. Higher shoot FW could be attributed to higher root FW since larger root systems improved nutrient and water uptake (He et al., 2001; Dodd, 2005), under such warm A-RZ conditions. Although the RIL had higher root/shoot ratios (Figure 2C) than the domesticated cultivar at all RZTs, respectively, this conferred no obvious benefits in terms of yield (i.e., shoot biomass) at A-RZT but instead only at C-RZT.

Highest mortality was observed in *L. serriola* at high A-RZT (Figure 1B), and high ambient day/night temperature in a tropical greenhouse, probably since it is a wild-type drought-tolerant winter annual (Werk and Ehleringer, 1985). Such mortality has been rather consistently observed across many batches of experiments (results not shown). As *L. serriola* is a wild type that is well-adapted to desert conditions of hot-day-cool-nights, its relatively higher mortality could have been due to the lack of cooler “night” temperatures that aided recovery from heat stress (Xue et al., 2011), evident from the highest accumulated RZ ethylene concentrations at A10-RZ treatment (Figure 5).

Lower SLA, implying thicker leaves (Chatterjee and Solankey, 2015), was observed for RIL and *L. serriola* at higher tropical A-RZT than their corresponding C-RZT (Figure 2D)—the thickest leaves were measured under A10-RZ treatment. Conversely, “Salinas” had the thinnest leaves at this same treatment. Likewise, Dale (1965) reported thinner leaves of *Phaseolus vulgaris* at warmer growing temperatures of 25°C than at 15°C. As such, this could be a strategy adopted by “Salinas” to increase surface area for increased thermal dissipation since there was correspondingly lowest shoot growth at A10 (Figure 2A). On the other hand, lower SLA (i.e., thicker leaves) occurred at higher growing temperatures in tomatoes (Abdelmageed et al., 2009). For C5 and C10, the lower shoot FW and thicker leaves (Figures 2A,D) observed in all genotypes could be attributed to the 4 h period during which C10-RZT exceeded 25°C (Figure 1A). “Salinas” also had thicker leaves when grown in C10, than C5 (Figure 2D)—opposite to that observed under A-RZ treatments. Decreased SLA, in comparison with cooler optimal growing temperatures, may be an alternative adaptive mechanism for reducing leaf area and increasing water use efficiency (Craufurd et al., 1999; Chatterjee and Solankey, 2015), to cope with thermal stress.

Chatterjee and Solankey (2015) attributed lower SLA and thicker leaves to higher density of Chl and other proteins per unit area. However, there was no significant difference in the total Chl content between the RZ treatments of A5 and A10, and C5 and C10 (Figure 3B). In comparing individual quantities of Chl a and Chl b (data not shown), significantly higher amounts of Chl b was obtained for the A10-RZ treatment only for the RIL and *L. serriola*. As Chl b protects the photosystem II reaction center from photodamage (Sakuraba et al., 2010), the higher Chl b concentration indicates the plants in A10-RZ treatment were rather stressed. However, lower Chl a/b ratios (Figure 3A) corresponded with highest shoot FWs (Figure 2A), at C5-RZ treatment, for all genotypes. As such, it is worthy to note that only extremely low Chl a/b ratios demonstrate that the plants were stressed (Dinç et al., 2012). Since carotenoids are also photoprotective in function (Filella et al., 2009), the Chl/carotenoid ratio better indicates plant stress (Hendry and Price, 1993). In this experiment, the Chl/carotenoid ratios are consistently lower in the A10 and C10 RZ treatments across all genotypes. For *L. serriola*, there were no significant differences in total Chl and carotenoid content across the RZ treatments but the Chl/carotenoid ratio was still the significantly lowest at A10 (Figure 3). Thus, this further demonstrates that this wild-type parent plant was growing under stressed conditions (Cui et al., 2006) in A10-RZ treatment.

NPQ, which reflects energy dissipated as heat, was significantly higher for the RIL in A10 and C10, than A5 and C5 (Figure 4A), indicating a higher ability to dissipate excess excitation energy (He, 2009) when misted less frequently. Comparing with the significantly lower qP and ETR values (Figures 4B,C) at A10 and C10 for the RIL, it demonstrates that less frequent misting has compromised photosynthetic function, and thus growth (Figure 2A), of these plants. In the case of *L. serriola*, NPQ was significantly lower at A10 and C10 instead (Figure 4A), with the C10 treatment significantly lower than A10. This drought tolerant wild-type *L. serriola* demonstrates its tolerance to high light, abundant in the tropics, despite the decreased frequency in irrigation. Though *L. serriola* had highest qP and ETR values (Figures 4B,C) at A10 and C10 RZ treatments, it had much lower shoot FW (Figure 2A) seeming to have invested its photosynthetic products into building a larger root system (Figure 2B) especially at C10 whilst sacrificing its shoot growth. “Salinas” most interestingly demonstrates significantly higher NPQ, qP and ETR (Figure 4) at A10 whilst growth is slower than at A5 and C5 RZ treatments. This could suggest that it reallocated its photosynthetic products to repair thylakoid membranes damaged by the combination of high light, typically found in a tropical greenhouse, and lowered misting frequency.

In this experiment, higher concentrations of RZ ethylene accumulated in A-RZT than C-RZT indicating greater plant stress (Abeles et al., 1992; Morgan and Drew, 1997; Lin et al., 2009) in the absence of RZ cooling. The highest RZ ethylene per unit root FW accumulated in A10-RZ treatment for *L. serriola* was doubles that of the highest values of “Salinas” and the RIL. Though it had significantly lower NPQ and higher photosynthetic performance than its counterparts in A5-RZ treatment (Figure 4), its shoot FW (Figure 2A) was also the lowest. In contrast, lowest RZ ethylene accumulated in C5-RZ treatment for the RIL (Figure 5) with correspondingly high photosynthetic rates (Figures 4B,C) which resulted in the high shoot FW (Figure 2A) and conversely low root/shoot ratio (Figure 2D). As such, these observations of higher photosynthetic rates and lower shoot FW and inversely high root/shoot ratio (Figures 2A,D) of *L. serriola* at A10-RZ treatment suggest that photosynthates have probably been redirected to stress recovery mechanisms.

Since shoot FW is the part of the plant with commercial value, and lower accumulated RZ ethylene concentrations corresponded with higher shoot growth at cooler RZTs, cultivars that are less RZT sensitive could be selected for agricultural purposes. As such, high electrical usage could be reduced with decreased misting frequency and/or chilling, decreasing production costs. Furthermore, root morphological analysis could be carried out to examine how the increase in RZ ethylene could affect root growth, since root systems support the shoots (He et al., 2001; Dodd, 2005) and would thus ultimately affect the commercially significant shoot FW.

## Author contributions

JH and SL initiated and funded the project. JH, ID, and TC planned the experiment. TC carried out the experiment and wrote the manuscript. JH and ID contributed ideas and improved the manuscript. All authors approved the manuscript.

## Funding

This project was funded by Singapore Millennium Foundation, Singapore.

### Conflict of interest statement

The authors declare that the research was conducted in the absence of any commercial or financial relationships that could be construed as a potential conflict of interest.

## Abbreviations

A: ambient
ACC: 1-aminocyclopropane-1-carboxylic acid
C: cool
Chl: chlorophyll
FW: fresh weight
RIL: recombinant inbred line
RZ: rootzone
RZT: rootzone temperature
SLA: specific leaf area.

## References

[B1] Abdelmageed A. H. A. Gruda N. El-Balla M. M. A. (2009). Performance of different tomato genotypes in the arid tropics of Sudan during the summer season. I. Vegetative growth. J. Agric. Rural De. Trop. Subtrop. 110, 137–145.

[B2] Abeles F. B. Morgan P. W. Salveit M. E. (1992). Ethylene in Plant Biology. San Diego, CA: Academic Press.

[B3] Ainscough E. W. Brodie A. M. Wallace A. L. (1992). Ethylene – an unusual plant hormone. J. Chem. Educ. 69, 315–318. 10.1021/ed069p315

[B4] Argyris J. Dahal P. Truco M. J. Ochoa O. Still D. W. Michelmore R. W. (2008). Genetic analysis of lettuce seed thermoinhibition. Acta Hortic. 782, 23–34. 10.17660/ActaHortic.2008.782.1

[B5] Argyris J. Truco M. J. Ochoa O. Knapp S. J. Still D. W. Lenssen G. M.. (2005). Quantitative trait loci associated with seed and seedling traits in *Lactuca*. Theor. Appl. Genet. 111, 1365–1376. 10.1007/s00122-005-0066-4 16177902

[B6] Barker D. H. Adams W. W. III. Demmig-Adams B. Logan B. A. Verhoeven A. S. Smith S. D. (2002). Nocturnally retained zeaxanthin does not remain engaged in a stage primed for energy dissipation during the summer in two Yucca species growing in the Mojave Desert. Plant Cell Environ. 25, 95–103. 10.1046/j.0016-8025.2001.00803.x

[B7] Berry J. A. Raison J. K. (1981). Responses of macrophytes to temperature, in Encyclopedia of Plant Physiology, eds Lange O. L. Nobel P. S. Osmond C. B. Ziegler H. (Heidelberg: Springer-Verlag), 277–338.

[B8] Björkman O. Powles S. B. (1984). Inhibition of photosynthetic reactions under water stress: interaction with light level. Planta 161, 490–504. 10.1007/BF00407081 24253918

[B9] Chatterjee A. Solankey S. S. (2015). Functional physiology in drought tolerance of vegetable crops: an approach to mitigate climate change impact, in Climate Dynamics in Horticultural Science, Vol. 1: The Principles and Applications, eds Choudhary M. L. Patel V. B. Siddiqui M. W. Mahdl S. S. (Boca Raton, FL: CRC Press), 149–171.

[B10] Choong T. W. (1998). Effects of Rootzone Temperature and Irradiance on the Growth and Photosynthetic Characteristics of Certain Subtropical Vegetable Crops, Honours theses, Nanyang Technological University.

[B11] Choong T. W. He J. Qin L. Dodd I. C. (2013). Identifying heat-resistant recombinant inbred lines (RILs) of lettuce in the tropics: productivity and root phenotyping. Acta Hortic. 1004, 173–180. 10.17660/ActaHortic.2013.1004.20

[B12] Craufurd P. C. Wheeler T. R. Ellis R. H. Summerfield R. J. Williams J. H. (1999). Effect of temperature and water deficit on water use efficiency, carbon isotope discrimination and specific leaf area in peanut. Crop Sci. 39, 136–142. 10.2135/cropsci1999.0011183X003900010022x

[B13] Cui L. Li J. Fan Y. Xu S. Zhang Z. (2006). High temperature effects on photosynthesis, PSII functionality and antioxidant activity of two *Festuca arundinacea* cultivars with different heat susceptibility. Bot. Stud. 47, 61–69.

[B14] Dale J. E. (1965). Leaf growth in Phaseolus vulgaris II. Temperature effects and the light factor. Ann. Bot. 29, 293–307.

[B15] Dinç E. Ceppi M. G. Tóth S. Z. Bottka S. Schansker G. (2012). The chl a fluorescence intensity is remarkably insensitive to changes in the chlorophyll content of the leaf as long as the chl a/b ratio remains unaffected. Biochim. Biophys. Acta 1817, 770–779. 10.1016/j.bbabio.2012.02.003 22342617

[B16] Dodd I. C. (2005). Root-to-shoot signalling: assessing the roles of ‘up’ in the up and down world of long-distance signalling *in planta*. Plant Soil 274, 251–270. 10.1007/s11104-004-0966-0

[B17] Douglas J. S. (1985). Advanced Guide to Hydroponics. London: Pelham Books.

[B18] Durst C. E. (1929). Inheritance in lettuce. Science 69, 553–554. 10.1126/science.69.1795.553 17818008

[B19] Ferakova V. (1976). *Lactuca* L, in Flora Europaea, Vol. 4, eds Tutin T. G. Heywood V. H. Burges N. A. Valentine D. H. (Cambridge, UK: Cambridge University Press), 328–331.

[B20] Filella I. Porcar-Castell A. Munne-Bosch S. Back J. Garbulsky M. F. Penuelas J. (2009). PRI assessment of long-term changes in carotenoids/chlorophyll ratio and short-term changes in de-epoxidation state of the xanthophyll cycle. Int. J. Remote Sens. 30, 4443–4455. 10.1080/01431160802575661

[B21] Freundl E. Steudle E. Hartung W. (1998). Water uptake by roots of maize and sunflower affects the radial transport of abscisic acid and its concentration in the xylem. Planta 207, 8–19. 10.1007/s004250050450

[B22] Harlan J. R. (1992). Crops and Man. Madison, WI: ACSESS Publications.

[B23] He C. J. Davies F. T. Jr, Lacey, R. E. (2009). Ethylene reduces gas exchange and growth of lettuce plants under hypobaric and normal atmospheric conditions. Physiol. Plant. 135, 258–271. 10.1111/j.1399-3054.2008.01190.x 19175518

[B24] He J. (2009). Impact of RZT on photosynthetic efficiency of aeroponically grown temperate and subtropical vegetable crops in the tropics, in Photosynthesis, eds Buchner T. B. Ewingen N. H. (New York, NY: Nova Science Publishers, Inc.), 111–144.

[B25] He J. Lee S. K. (1998). Growth and photosynthetic characteristics of lettuce (*Lactuca sativa* L.) under fluctuating hot ambient temperatures with the manipulation of cool rootzone temperature. J. Plant Physiol. 152, 387–391. 10.1016/S0176-1617(98)80252-6

[B26] He J. Lee S. K. (2004). Photosynthetic utilization of radiant energy by temperate lettuce grown under natural tropical condition with manipulation of root-zone temperature. Photosynthetica 42, 457–463. 10.1023/B:PHOT.0000046166.29815.94

[B27] He J. Lee S. K. Dodd I. C. (2001). Limitations to photosynthesis of lettuce grown under tropical conditions: alleviation by rootzone cooling. J. Exp. Bot. 52, 1323–1330. 10.1093/jexbot/52.359.1323 11432951

[B28] He J. Qin L. Liu Y. Choong T. W. (2015). Photosynthetic capacities and productivity of indoor hydroponically grown *Brassica alboglabra* Bailey under different light sources. Am. J. Plant Sci. 6, 554–563. 10.4236/ajps.2015.64060

[B29] He J. Tan B. H. G. Qin L. (2011). Source-to-sink relationship between green leaves and green pseudobulbs of C_3_ orchid in regulation of photosynthesis. Photosynthetica 49, 209–218. 10.1007/s11099-011-0023-1

[B30] He J. Tan L. P. Lee S. K. (2009). Rootzone temperature effects on photosynthesis, ^14^C-photoassimilate partitioning and growth of temperate lettuce (*Lactuca sativa* cv. ‘Panama’) in the tropics. Photosynthetica 47, 95–103. 10.1007/s11099-009-0015-6

[B31] Hendry G. A. F. Price A. H. (1993). Stress indicators: chlorophylls and carotenoids, in Methods in Comparative Plant Ecology, eds Hendry G. A. F. Grime J. P. (London: Chapman & Hall), 148–152.

[B32] Jeffrey C. (1975). *Lactuca* L, in Flora of Turkey and the east Aegean islands, Vol. 5, eds Davis P. H. Mill R. R. Tan K. (Edinburgh: Ediniburgh University Press), 776–782.

[B33] Kaspar T. C. Bland W. L. (1992). Soil temperature and root growth. Soil sci. 154, 290–299. 10.1097/00010694-199210000-00005

[B34] Kesseli R. V. Ochoa O. Michelmore R. W. (1991). Variation at RFLP loci in *Lactuca* spp. and origin of cultivated lettuce (*L. sativa*). Genome 34, 430–436. 10.1139/g91-065

[B35] Kirpicznikov M. E. (1964). *Lactuca* L, in Komarov's Flora of URSS, Vol. 29, ed Komarov V. L. (Nuaka: Moscow-Leningrad), 274–317.

[B36] Lee S. K. (1993). Aeroponic system as a possible alternative for crop production in Singapore. Commonwealth Agric. Digest 3, 1–4.

[B37] Lee S. K. Wong Y. W. Liu C. Y. Cheong S. (1994). Optimising aeroponic systems for urban farming in Singapore, in Paper Presented at the International Conference on Advances in Tropical Agriculture in the Twentieth Century and Prospects for the Twenty-First Century (Trinidad: University of the West Indies).

[B38] Lee S. Reid D. (1997). The role of endogenous ethylene in the expansion of *Helianthus annuus* leaves. Can. J. Bot. 75, 501–508. 10.1139/b97-054 11541081

[B39] Lin Z. Zhong S. Grierson D. (2009). Recent advances in ethylene research. J. Exp. Bot. 60, 3311–3336. 10.1093/jxb/erp204 19567479

[B40] Morgan P. W. Drew M. C. (1997). Ethylene and plant responses to stress. Plant Physiol. 100, 620–630. 10.1111/j.1399-3054.1997.tb03068.x

[B41] Pallaghy C. K. Raschke K. (1972). No stomatal response to ethylene. Plant Physiol. 49, 275–276. 10.1104/pp.49.2.275 16657942 PMC365946

[B42] Pierik R. Tholen D. Poorter H. Visser E. J. W. Voesenek A. C. J. (2006). The Janus face of ethylene: growth inhibition and stimulation. Trends Plant Sci. 11, 176–183. 10.1016/j.tplants.2006.02.006 16531097

[B43] Qin L. He J. Lee S. K. Dodd I. C. (2007). An assessment of ethylene mediation of lettuce (*Lactuca sativa*) root growth at high temperatures. J. Exp. Bot. 58, 3017–3024. 10.1093/jxb/erm156 17728295

[B44] Ruzicka K. Ljung K. Vanneste S. Podhorska R. Beeckman T. Friml J.. (2007). Ethylene regulates root growth through effects on auxin biosynthesis and transport-dependent auxin distribution. Plant Cell 19, 2197–2212. 10.1105/tpc.107.052126 17630274 PMC1955700

[B45] Sakamoto M. Suzuki T. (2015). Effect of rootzone temperature on growth and quality of hydroponically grown red leaf lettuce (*Lactuca sativa* L. cv. Red Wave). Am. J. Plant Sci. 6, 2350–2360. 10.4236/ajps.2015.614238

[B46] Sakuraba Y. Yokono M. Akimoto S. Tanaka R. Tanaka A. (2010). Deregulated chlorophyll b synthesis reduces the energy transfer rate between photosynthetic pigments and induces photodamage in *Arabidopsis thaliana*. Plant Cell Physiol 51, 1055–1065. 10.1093/pcp/pcq050 20403808

[B47] Salisbury F. B. Ross C. W. (1992). Plant Physiology. Belmont, CA: Wadsworth, Inc.

[B48] Tachibana S. (1982). Comparison of effects of root temperature on the growth and mineral nutrition of cucumber cultivars and figleaf gourd. J. Jpn. Soc. Hortic. Sci. 51, 299–308. 10.2503/jjshs.51.299

[B49] Thompson H. C. Langhans R. W. Both A. J. Albright L. D. (1998). Shoot and root temperature effects on lettuce growth in a floating hydroponic system. J. Amer. Soc. Hortic. Sci. 123, 361–364.

[B50] Verhoeven A. S. Demmig-Adams B. Adams W. W. III. (1997). Enhanced employment of the xanthophyll cycle and thermal energy dissipation in spinach exposed to high light and N stress. Plant Physiol. 113, 817–824. 12223645 10.1104/pp.113.3.817 PMC158201

[B51] Wellburn A. R. (1994). The spectral determination of chlorophylls a and b, as well as carotenoids, using various solvents with spectrophotometers of different resolution. J. Plant Physiol. 144, 307–313. 10.1016/S0176-1617(11)81192-2

[B52] Werk K. S. Ehleringer J. (1985). Photosynthetic characteristics of *Lactuca serriola* L. Plant Cell Environ. 8, 345–350. 10.1111/j.1365-3040.1985.tb01409.x

[B53] Woodrow L. Grodzinski B. (1989). An evaluation of the effects of ethylene on carbon assimilation in *Lycopersicon esculentum* Mill. J. Exp. Bot. 40, 361–368. 10.1093/jxb/40.3.361

[B54] Woodrow L. Jiao J. Tsujita L. Grodzinski B. (1989). Whole plant and leaf steady state gas exchange during ethylene exposure in *Xanthium strumarium*. Plant Physiol. 90, 85–90. 10.1104/pp.90.1.85 16666773 PMC1061681

[B55] Xue W. Li X. Y. Lin L. S. Wang Y. J. Li L. (2011). Effects of elevated temperature on photosynthesis in desert plant *Alhagi sparsifolia* S. Photosynthetica 49, 435–447. 10.1007/s11099-011-0054-7

[B56] Zohary D. (1991). The wild genetic resources of cultivated lettuce (*Lactuca sativa* L.). Euphytica 53, 31–35. 10.1007/BF00032029

